# Dung Beetle Community and Functions along a Habitat-Disturbance Gradient in the Amazon: A Rapid Assessment of Ecological Functions Associated to Biodiversity

**DOI:** 10.1371/journal.pone.0057786

**Published:** 2013-02-27

**Authors:** Rodrigo F. Braga, Vanesca Korasaki, Ellen Andresen, Julio Louzada

**Affiliations:** 1 Universidade Federal de Lavras, Departamento de Biologia, Setor de Ecologia e Conservação Campus Universitário, Lavras, Minas Gerais, Brazil; 2 Centro de Investigaciones en Ecosistemas, Universidad Nacional Autónoma de México, Campus Morelia, Michoacán, México; Université Paris 13, France

## Abstract

Although there is increasing interest in the effects of habitat disturbance on community attributes and the potential consequences for ecosystem functioning, objective approaches linking biodiversity loss to functional loss are uncommon. The objectives of this study were to implement simultaneous assessment of community attributes (richness, abundance and biomass, each calculated for total-beetle assemblages as well as small- and large-beetle assemblages) and three ecological functions of dung beetles (dung removal, soil perturbation and secondary seed dispersal), to compare the effects of habitat disturbance on both sets of response variables, and their relations. We studied dung beetle community attributes and functions in five land-use systems representing a disturbance gradient in the Brazilian Amazon: primary forest, secondary forest, agroforestry, agriculture and pasture. All response variables were affected negatively by the intensification of habitat disturbance regimes, but community attributes and ecological functions did not follow the same pattern of decline. A hierarchical partitioning analysis showed that, although all community attributes had a significant effect on the three ecological functions (except the abundance of small beetles on all three ecological functions and the biomass of small beetles on secondary dispersal of large seed mimics), species richness and abundance of large beetles were the community attributes with the highest explanatory value. Our results show the importance of measuring ecological function empirically instead of deducing it from community metrics.

## Introduction

The relevance of assessing ecological functions in conservation-oriented studies is becoming increasingly recognized [1], [2], [3], [4], [5]. This is because of a better understanding of the relations between biodiversity, ecological functions and ecosystem integrity, which has led to recognition of the many possible direct and indirect consequences of the imminent biodiversity crisis [1], [6]. Species ecological functions are often difficult to measure on a quantitative basis. Thus, the study of taxa that are conspicuous and important components of ecosystems is particularly promising in those cases in which not only the organisms, but also their ecological functions can be estimated using cost-effective approaches.

Dung beetles (Scarabaeidae: Scarabaeinae) are a diverse, abundant group of insects that have been extensively used as a cost-effective indicator taxon, particularly for studies focusing on the consequences of habitat disturbance [7], [8]. Dung beetles feed mainly on decomposing matter, mostly vertebrate feces, carrion, decaying fruits and fungi [9]. Studies have reported that dung removal and burial by dung beetles has many beneficial ecological consequences, such as soil fertilization and aeration [10], improved nutrient cycling and uptake by plants [11], increase in pasture quality [12], biological control of pest flies and intestinal parasites [13] and secondary seed dispersal [14]. Some of these ecological functions can be considered to be ecosystem services, because of their potentially large economic importance and positive impacts on human well-being [15], [16].

The popularity of dung beetles as a focal group is evident through a large and rapidly increasing list of published studies, which focus on the effects of various types of habitat disturbance on the composition and structure of dung beetle communities [17], [18], [19], [20]. Although many of these studies recognize the importance of distinguishing among functional groups or guilds of beetles (e.g. [21], [22]), few studies include measures of functional diversity in their analyses [23] or quantify the functions performed to relate them back to community aspects (e.g. [24], [25], [26]).

Here, we aimed to determine the effects of habitat disturbance on community attributes (richness, abundance, and biomass) as well as on dung beetle functions (dung removal, soil excavation and secondary seed dispersal), and to assess the predictive power of community attributes to describe the amount of ecological function.

## Materials and Methods

### Study Site

**Figure 1:**
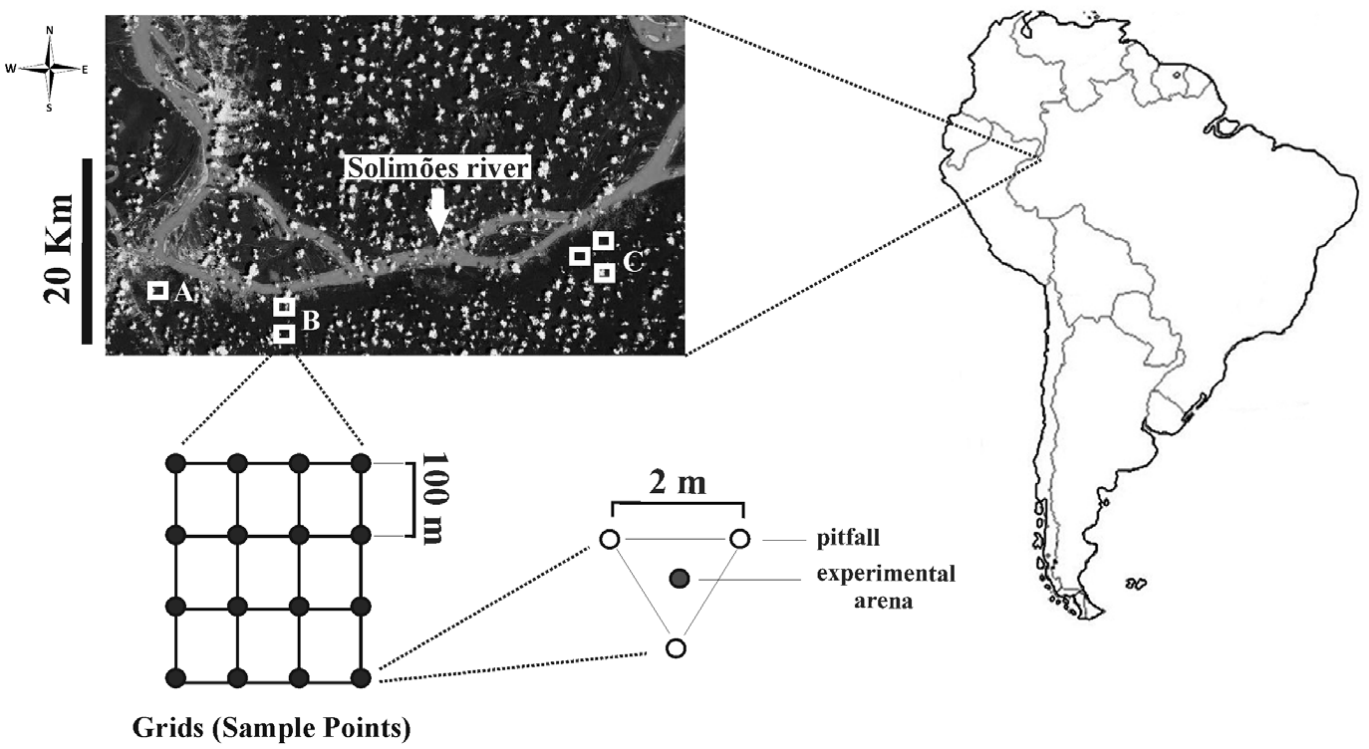
Study areas and sampling design in Amazonas State, Brazil (reproduced, with permission, from Google Earth™). (A) Benjamin Constant municipality. (B) Guanabara II community. (C) Noval Aliança community. Replicates of each land-use treatment were proportionally distributed inside the grids according to the availability of distinct land-use systems in the study area. Each sample point was comprised of three pitfall traps for the measurement of community attributes and one experimental dung unit for the measurement of functions [31].

The study was carried out during March 2008 in the municipality of Benjamin Constant (4°21′12′′S and 69°36′04′′W, and 4°25′37′′S and 69°54′23′′W), in the Brazilian state of Amazonas, near the border between Brazil, Colombia and Peru. The study sites included the communities of Guanabara II, Nova Aliança and the town of Benjamin Constant (Figure 1).

The regional climate is classified as humid–super humid Af (Köppen), with a mean annual temperature and rainfall of 25.7°C and 2,562 mm, respectively. No pronounced dry season occurs, with precipitation in the driest month being >100 mm; the wettest period is December–April. Inceptisol is the dominant soil class in the region [27].

The study was conducted as part of a larger, multinational research project (“Conservation and Sustainable Management of Below-Ground Biodiversity”). In total, 73 sampling points were distributed across six grids, approximately 9 ha each, to include all the major regional land-use systems (LUSs) (Figure 1). Each sampling point was geo-referenced and was part of a pre-established grid, following the institutional norms of the project [28]. The distance between points was generally 100 m, but was reduced to 50 m in some cases where more replicates per LUS were necessary, ensuring the independence of dung beetle samples [29]. We evaluated five LUSs that, from least to most disturbed habitat were: primary forest (n = 15) representing the original forest cover; secondary forest (n = 14) 5–15 years after abandonment of shifting cultivation plots; agroforest (n = 15), which represented forest that had never been cleared, but in which some selective logging had occurred with subsequent planting of several commercial species underneath a canopy of native trees; agriculture (n = 14) small-scale slash and burn shifting cultivation, plots <1.5 ha with annual (cassava, corn, sugar cane and pineapple) and semi-perennial crops (banana); and, pasture (n = 15 ) which included areas for livestock planted in 1970 with imperial grass (*Axonopus scoparius*), after which it was substituted with *Brachiaria brizantha*, *Brachiaria humidicola* and *Paspalum notatum*. Replicates of each land-use treatment were proportionally distributed inside the grids according to the availability of distinct land-use systems in the study area.

### Dung Beetle Community Attributes

To quantify beetle species richness, abundance and biomass, we collected beetles using baited pitfall traps. In all sampling points, we placed three baited pitfall traps (19-cm diameter, 11-cm deep), one trap in each corner of a 2-m-side triangle. We used three traps per sampling point to maximize the number of captures and minimize the consequences of potential trap loss. However, in situations in which dung availability or other research resources are limiting, a single pitfall trap can be used. Traps contained 250 ml of a salt+detergent solution, and were baited with fresh human dung (25 g). Traps were opened in the morning and captured beetles were collected after 24 h. We decided to use a 24 h period, rather than the more commonly used 48 h sampling period, to minimize the effects of a confounding factor, i.e. dung attractiveness. It is well known that as dung dries out, it quickly loses its attractiveness [30]. As the land-use systems being compared most likely varied in terms of the speed with which the dung lost attractiveness, a 24 h period was chosen to minimize such differences. All specimens were preserved and sent to the Invertebrate Ecology and Conservation Laboratory, at the Universidade Federal de Lavras (UFLA) where all individuals were sorted and identified. To obtain body mass estimates and size for each species, a sample of 1–30 individuals was dried at 40°C to constant weight, and weighed in a 0.0001 g precision balance [31]. The length from the clypeus to the pygidium was measured for each species using calipers as a proxy for dung beetle species size. The number of individuals of each species used for biomass and size estimation varied according to the number of beetles available. All necessary permits were obtained for the described field studies. Responsible for the authorization: Ministério do Meio Ambiente (MMA); Instituto Brasileiro do Meio Ambiente e dos Recursos Naturais Renováveis (IBAMA); and Sistema de Autorização e Informação em Biodiversidade (SISBIO); license number 10061-1. Authentication code: 11933184; http://www.icmbio.gov.br/sisbio/verificar-autenticidade.html.

### Dung Beetle Community Functions

**Figure 2:**
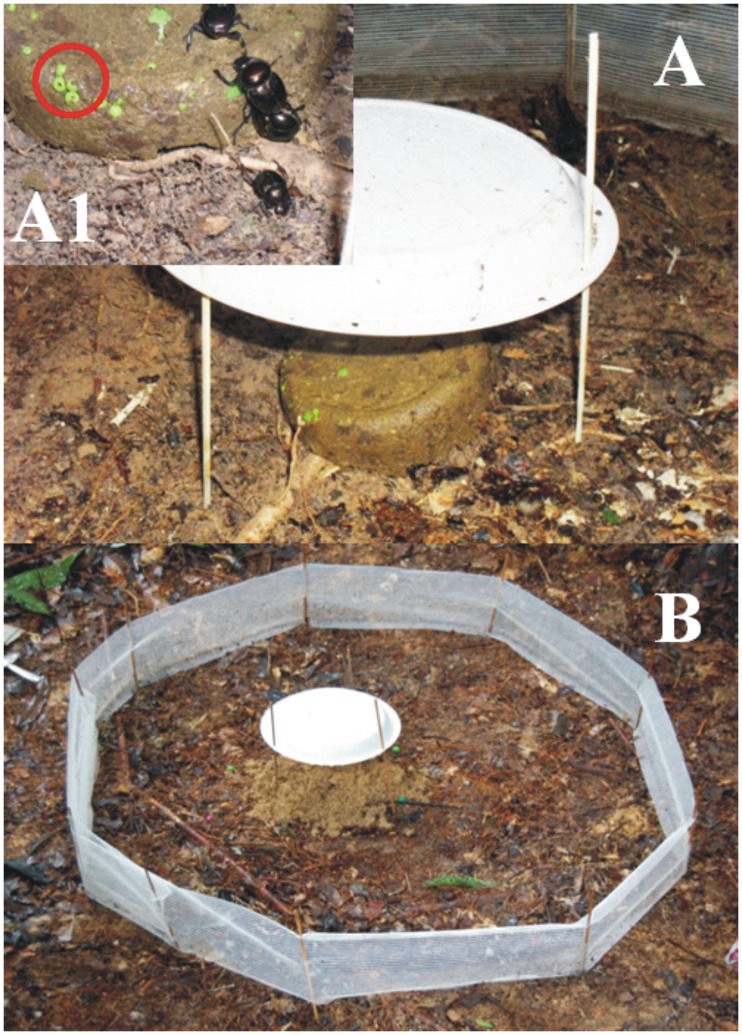
Experimental arena used for measuring three ecological functions of dung beetles. (A) The experimental dung pile in the center of the arena should be protected from rain. (A1) Plastic beads of different sizes were placed within the dung as seed mimics. (B) General setup of the experimental arena (see main text for description).

We set up an ecological functions experiment the day before the dung beetles were sampled. The sampling protocol consisted of establishing a circular plot, 1 m in diameter, the border of which was delimited by a fence (approximately 15 cm high; Figure 2). We built the fence using a nylon net with a mesh size of 0.08 mm, which was held in place by eight bamboo sticks. The fence limited the horizontal movement of dung portions by dung beetles to a contained area, allowing for a more accurate quantification of functions. To further facilitate the measurement of ecological functions, we first cleared the soil surface of each arena of litter and vegetation.

In the center of each arena we placed an experimental dung pile consisting of 70 g of a mixture of equal proportions of fresh human and swine dung. Human and pig dung are similar to the dung of the most abundant mammal dung suppliers active in the region, namely monkeys and peccaris. Furthermore, human feces have been shown to be an effective attractant to a wide range of dung beetle species [29].

Inside each experimental dung pile we placed plastic beads, used as seed mimics, in order to estimate the function of secondary seed dispersal. Plastic beads have been used as seed mimics successfully in various studies assessing seed dispersal by dung beetles (e.g. [32], [21]). No significant differences have been found in the rate or depth of burial between seeds or seed mimics [33] and seeds mimics have the great advantage of not being removed by seed predators [25]. In each experimental dung pile, we placed seed mimics of three sizes: 50 small seeds (3.5-mm diameter), 20 medium seeds (8.6-mm diameter) and 10 large seeds (15.5-mm diameter). We protected each experimental dung pile from direct rain by placing a small plastic plate above it, as roof (Figure 2).

Ecological functions were measured 24 h after the placement of the experimental dung piles [31], to allow for the activity of both diurnal and nocturnal dung beetles. We weighed the dung remaining on the soil surface and in excavated soil. All seed mimics still present in the remaining dung were removed, counted and weighed. The weight of seed mimics was subtracted from the dung weight to obtain the net amount of dung remaining and then the amount of dung removed by beetles was calculated. To quantify the amount of soil excavated by dung beetles (i.e. soil moved from deep layers onto the surface as a consequence of tunnel building), loose soil (clearly identifiable on the surface) was collected with spoons or spatulas and dried at 100°C until it reached a constant weight. Although ecological functions might be underestimated due to changes in competitive interactions caused by the fence, we believe that this method provides good estimated values for the amount of functions performed.

To quantify seed dispersal, all seed mimics not found in the dung remaining on the soil surface were assumed to have been dispersed by dung beetles. Thus, the number of seed mimics dispersed was obtained by subtracting the number of seed mimics in the remaining dung from the number of seed mimics originally mixed in the dung pile. It is important to mention that this methodology assesses one component of the dispersal effectiveness (*sensu* [34]) provided by dung beetles, namely the quantity component. Aspects of dispersal quality, such as burial depth, could be assessed by marking some of the beads with a 50-cm long thread (see [35] for details).

### Data Analysis

We assessed the sampling efficiency of each LUS by calculating the number of observed species as a percentage of the total species richness, which was estimated based on the average of three nonparametric estimators: Chao 1, Jack 1 and Bootstrap [36]. Community attributes (abundance, richness and biomass) were estimated for each sampling point, both for the total dung beetle assemblage captured, and for the small- and large-beetle assemblages separately (hereafter referred to as ‘total’, ‘small’ and ‘large’). This was done because large dung beetles are known to be responsible for a large proportion of the ecological functions performed by the community [51]. We defined the small- and large-beetle assemblages as proposed by [37], with species <10 mm in length constituting the former and species ≥10 mm the latter.

To analyze the effects of land use on dung beetle community response variables, we used generalized linear models (GLMs), with land-use categories (primary forest, secondary forest, agroforest, agriculture and pasture) as fixed factors. Data from the three pitfall traps placed in each sampling point were pooled, because our sampling unit was the sampling point (n = 15 for all systems except agriculture, where n = 14). We used a Poisson error structure for beetle abundance (total, large and small), richness (total, large and small) and biomass (total), a quasi-Poisson error structure when overdispersion was detected and a binomial error for proportion data (seed dispersal and dung removal) or quasi-Binomial error when overdispersion was detected [38]. We used Gaussian error structure for soil excavation. All GLMs were checked with residual analyses to evaluate the adequacy of the error distribution [39]. To test for correlation between dung removal and the other functions (soil excavation and seed dispersal), we calculated Spearman correlation coefficients.

We used hierarchical partitioning [40] to examine the independent effects of six predictive variables derived from the combination of the three community attributes (richness, abundance and biomass) and the two components of the total beetle assemblage (small and large) on the ecological functions of dung beetles (dung removal, soil excavated and seed dispersal). Hierarchical partitioning is a multiple-regression technique in which all possible linear models are jointly considered to identify the most likely causal factors, providing a measure of the effect of each variable that is largely independent from effects of other variables [40], [41]. We evaluated competing models based on the *R* ^2^_dev_ statistic, determining the significance of effects with a randomization test with 500 interactions [42]. Hierarchical partitioning and associated randomization tests were implemented using the hier.part package freely available in the R statistical program [38].

## Results

We captured 1159 dung beetles representing 45 species (Table 1). Primary forest was the land-use system with the highest number of species recorded (33), followed by secondary forest (17), agroforest (16), agriculture (13) and pasture (3). In primary forest, we captured 786 individuals, whereas dung beetles were all but absent from most pitfall traps in the pasture, with only six individuals captured; the number of individuals in the other three land-use systems ranged from 85 to 188 (Table 1). Sampling efficiency ranged from approximately 66% in secondary forest to 83% in primary forest.

**Table 1:** Total number of individuals, by species, captured in different land-use systems in Benjamin Constant, AM, Brazil.

Tribe/Species	Body size			Number of individuals captured					Total
	Mean weight (g)	*n*	Length category	PF	AF	SF	AG	PA	Mean
**ATEUCHINI**									
*Ateuchus* aff. *connexus* (Harold, 1868)	0.0146	2	Small	1	0	0	0	0	1
*Ateuchus* aff. *scatimoides* (Balthasar, 1939)	0.0080	33	Small	5	0	0	0	0	5
*Ateuchus* aff. *simplex* (Serville, 1828)	0.0182	11	Small	3	0	0	0	0	3
*Uroxys* sp. 1	0.0044	16	Small	2	0	0	0	0	2
*Uroxys* sp. 3	0.0027	7	Small	0	0	1	0	0	1
Ateuchini new genus	0.0072	2	Small	0	1	0	0	0	1
**CANTHONINI**									
*Anisocanthon* n. sp. 1	0.0103	3	Small	0	0	0	2	0	2
*Canthon* aff. *angustatus* Harold, 1867	0.0103	13	Small	2	0	0	0	0	2
*Canthon quadriguttatus* (Olivier, 1789)	0.0079	5	Small	0	1	0	0	0	1
*Canthon* aff. *quinquemaculatus* Castelnau, 1840	0.0577	16	Large	0	5	2	1	0	8
*Canthon mutabilis* Lucas, 1857	0.0106	31	Small	0	0	0	19	0	19
*Canthon proseni* (Martinez, 1949)	0.0949	31	Large	83	0	0	0	0	83
*Deltochilum amazonicum* Bates, 1887	0.4578	14	Large	1	0	1	0	0	2
*Deltochilum carinatum* (Westwood, 1837)	0.2465	3	Large	1	0	0	0	0	1
*Deltochilum* sp. 1	0.0953	25	Large	1	0	1	0	0	2
*Deltochilum* sp. 2	0.0760	14	Large	2	0	1	0	0	3
*Pseudocanthon* aff. *xanthurus* (Blanchard, 1845)	0.0030	30	Small	0	0	0	135	1	136
**COPRINI**									
*Canthidium* (*Canthidium*) aff. *depressum* (Boucomont, 1928)	0.0218	35	Small	1	1	1	0	0	3
*Canthidium* (*Canthidium*) sp. 1	0.0087	2	Small	13	0	0	0	0	13
*Dichotomius fortestriatus* (Luederwaldt, 1923)	0.0873	31	Large	70	0	0	0	0	70
*Dichotomius mamillatus* (Felsche, 1901)	0.4076	32	Large	14	0	0	0	0	14
*Dichotomius ohausi* (Luederwaldt, 1923)	0.1821	14	Large	5	0	0	0	0	5
*Dichotomius robustus* (Luederwaldt, 1935)	0.1076	3	Large	1	0	0	0	0	1
*Dichotomius* aff. *podalirius* Felshe, 1901	0.2972	31	Large	12	0	0	0	0	12
*Ontherus pubens* Génier, 1996	0.0731	31	Large	0	43	13	10	1	67
**ONITICELLINI**									
*Eurysternus strigilatus* Génier, 2009	0.0103	31	Small	9	1	7	0	0	17
*Eurysternus wittmerorum Martinez, 1988*	0.0229	31	Small	34	0	0	0	0	34
*Eurysternus caribaeus* (Herbst, 1789)	0.0867	31	Large	164	3	11	0	0	178
*Eurysternus hamaticollis* Balthasar, 1939	0.1980	32	Large	15	0	1	0	0	16
*Eurysternus howdeni* Génier, 2009	0.0179	28	Small	5	1	1	1	0	8
*Eurysternus foedus* Guérin-Ménéville, 1844	0.1327	32	Large	38	3	0	0	0	41
*Eurysternus hypocrita* Balthasar, 1939	0.1335	31	Large	148	3	6	1	0	158
**ONTHOPHAGINI**									
*Onthophagus* aff. *acuminatus* Harold, 1880	0.0066	31	Small	0	25	10	6	0	41
*Onthophagus* aff. *digitifer* Boucomont, 1932	0.0010	1	Small	1	0	0	0	0	1
*Onthophagus* aff. *marginicollis* Harold, 1880	0.0072	33	Small	0	3	0	1	4	8
*Onthophagus* aff. *xanthomerus* Bates, 1887	0.0109	6	Small	3	0	0	0	0	3
*Onthophagus* aff. *bidentatus* (Drapiez, 1819)	0.0124	33	Small	62	7	0	1	0	70
*Onthophagus* aff. *haematopus* Harold, 1875	0.0090	30	Small	46	2	1	0	0	49
**PHANAEINI**									
*Coprophanaeus telamon* (Erichson, 1847)	0.5143	31	Large	2	1	5	1	0	9
*Oxysternon conspicillatum* (Weber, 1801)	0.5516	6	Large	1	0	0	0	0	1
*Oxysternon lautum* (MacLeay, 1819)	0.2200	1	Large	0	1	0	0	0	1
*Oxysternon silenus peruanum* Pereira, 1943	0.1668	34	Large	13	11	23	7	0	54
*Phanaeus bispinus* Bates, 1868	0.1822	4	Large	0	0	0	3	0	3
*Phanaeus cambeforti* Arnaud, 1982	0.1176	14	Large	7	0	0	0	0	7
*Phanaeus chalcomelas* (Perty, 1830)	0.1580	18	Large	3	0	0	0	0	3
**Total number of individuals**				768	112	85	188	6	1159
**Total number of species**				33	17	16	13	3	45

Mean body weight of dung beetle species, the number of beetles used to calculate mean body weight (*n*) and body length category (‘Small’ for species <10 mm long and ‘Large’ for species ≥10 mm long) are also shown. Land-use systems are: primary forest (PF), agroforest (AF), secondary forest (SF), agriculture (AG) and pasture (PA).

**Figure 3:**
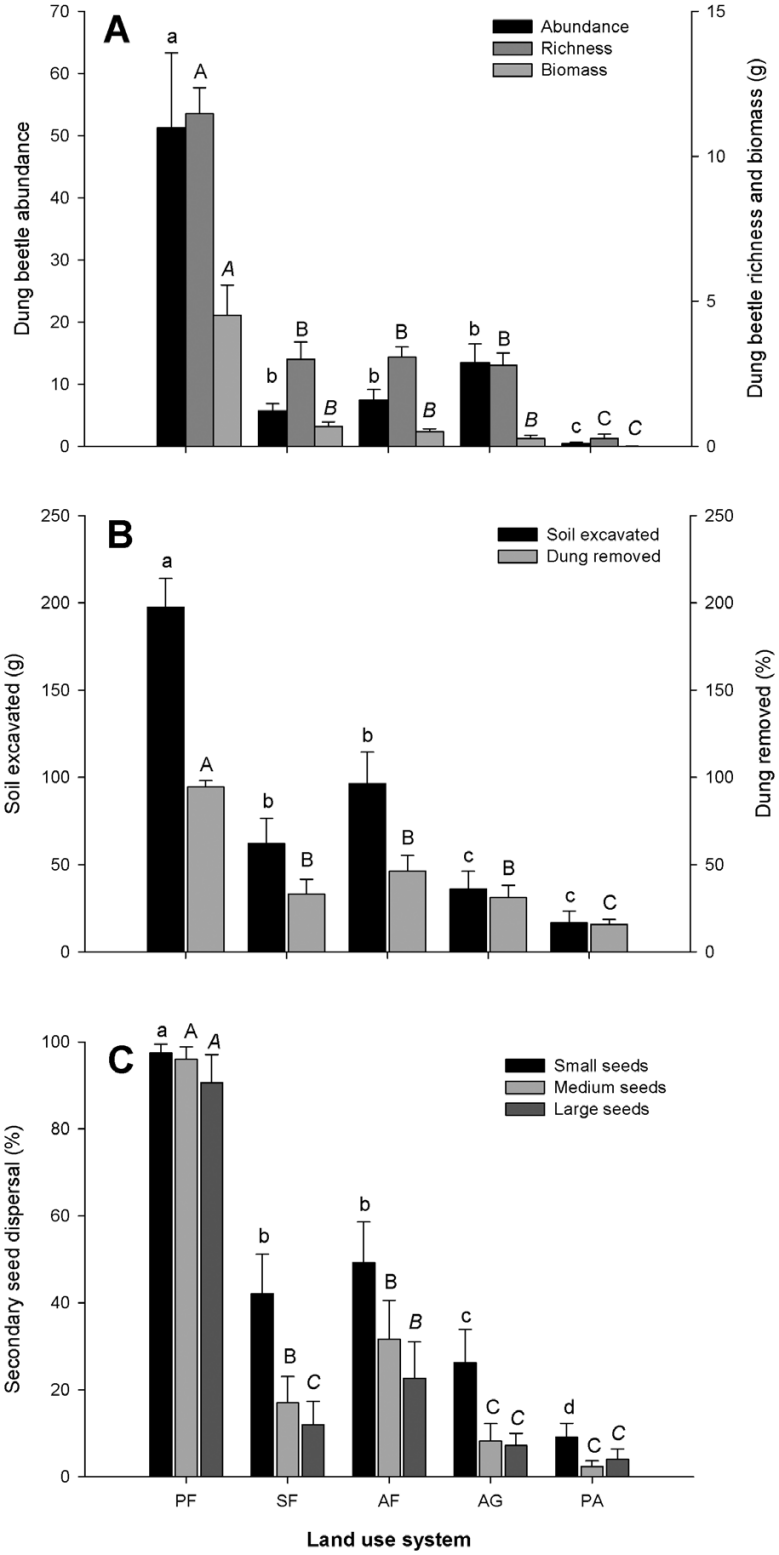
Mean values of (A) abundance, biomass and richness of dung beetles, (B) amount of soil excavated and dung removed, and (C) secondary dispersal of small, medium and large seed mimics. Land-use systems sampled were: primary forest (PF), secondary forest (SF), agroforest (AF), agriculture (AG) and pasture (PA). Different letters above bars indicate statistically significant differences (p<0.05) among land-use systems. Error bars represent ±1 SEM.

Mean dung beetle abundance (F_4,69_ = 26.78, p<0.001), richness (χ^2^ = 241.52, p<0.001) and biomass (F_4,69_ = 34.76, p<0.001) changed across the land-use intensification gradient (Figure 3A), with the highest values in primary forest and the lowest values in pasture. Agroforest, secondary forest and agriculture composed a statistically homogenous group for all community attributes (Figure 3A; Table S1).

**Figure 4:**
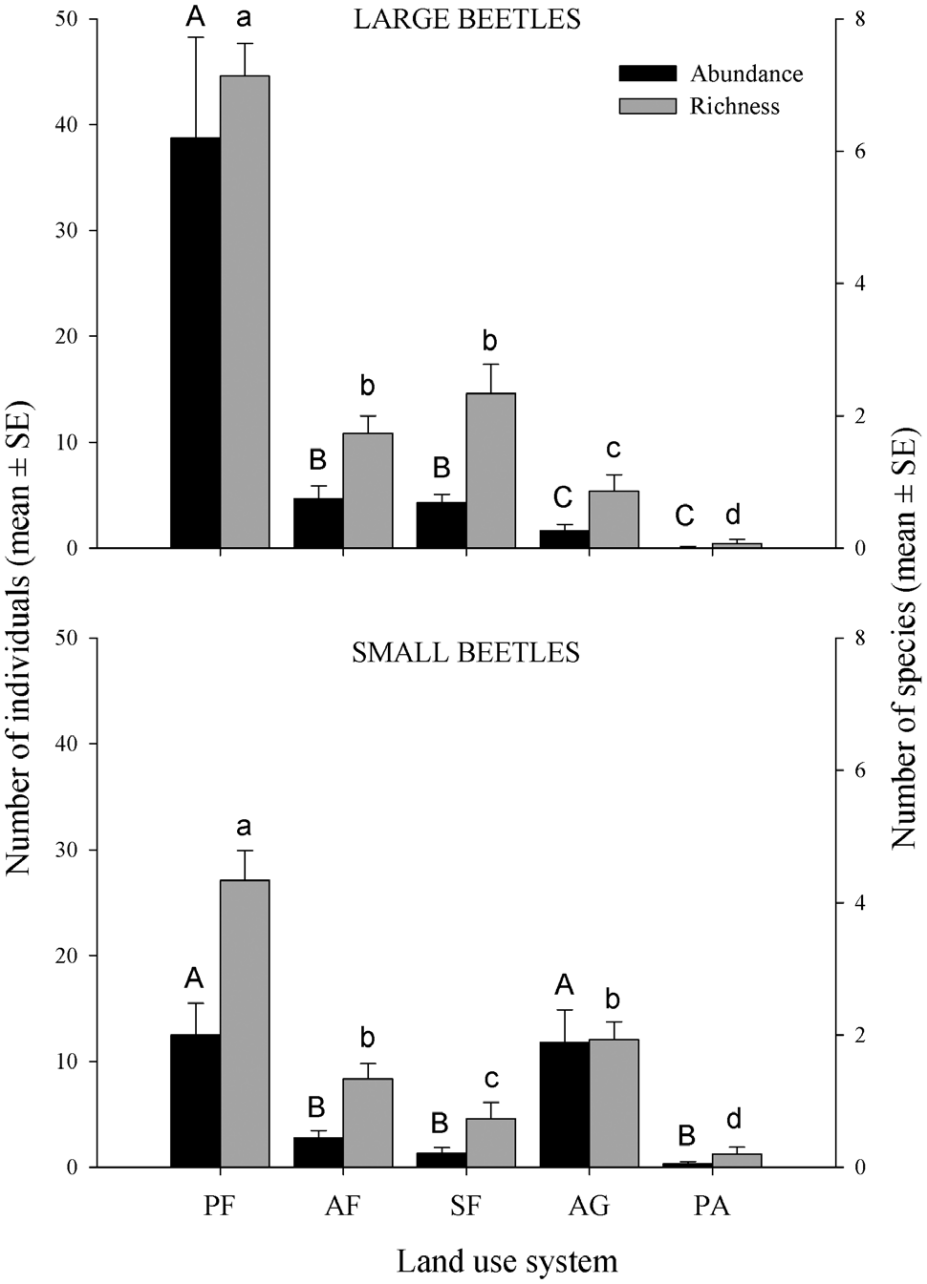
Mean values of large and small dung beetle abundance and richness. The land-use systems sampled were: primary forest (PF), secondary forest (SF), agroforest (AF), agriculture (AG) and pasture (PA). Different letters above bars indicate statistically significant differences (p<0.05) among land-use systems. Error bars represent ±1 SEM.

Abundance (F_2,70_ = 63.21; p<0.001) and richness (F_3,69_ = 62.76; p<0.001) of large beetles decreased with increasing land-use intensity (Figure 4). However, small beetles showed the same abundance in primary forest and agriculture (F = 0.04; p = 0.83) and these values were higher than those in the other systems (F = 56.77; p<0.001). Richness of small beetles was greatest in primary forest, followed by agriculture and agroforest, which showed no difference between them, then secondary forest, and finally pasture with the lowest richness (F_3,69_ = 34.63; p<0.001; Figure 4; Table S1).

All three ecological functions performed by dung beetles were negatively affected by land-use intensity (dung removal: F_4,69_ = 17.84, p<0.001; soil excavation: χ2_4,69_ = 64.71, p<0.001; dispersal of small seed mimics: F_4,69_ = 21.00, p<0.001; medium seeds mimics: F_4,69_ = 31.90, p<0.001 and large seed mimics: F_4,69_ = 22.51, p<0.001; Figure 3B,C; Table S1). As expected, both the amount of soil excavated and secondary seed dispersal were positively correlated with dung removal, as the former two functions are a direct consequence of the latter (correlations between dung removal and: soil excavation, Rs = 0.752, p<0.001; small seed mimic dispersal, Rs = 0.853, p<0.001; medium seed mimic dispersal, Rs = 0.839, p<0.001; and large seed mimic dispersal, Rs = 0.724, p<0.001).

**Figure 5:**
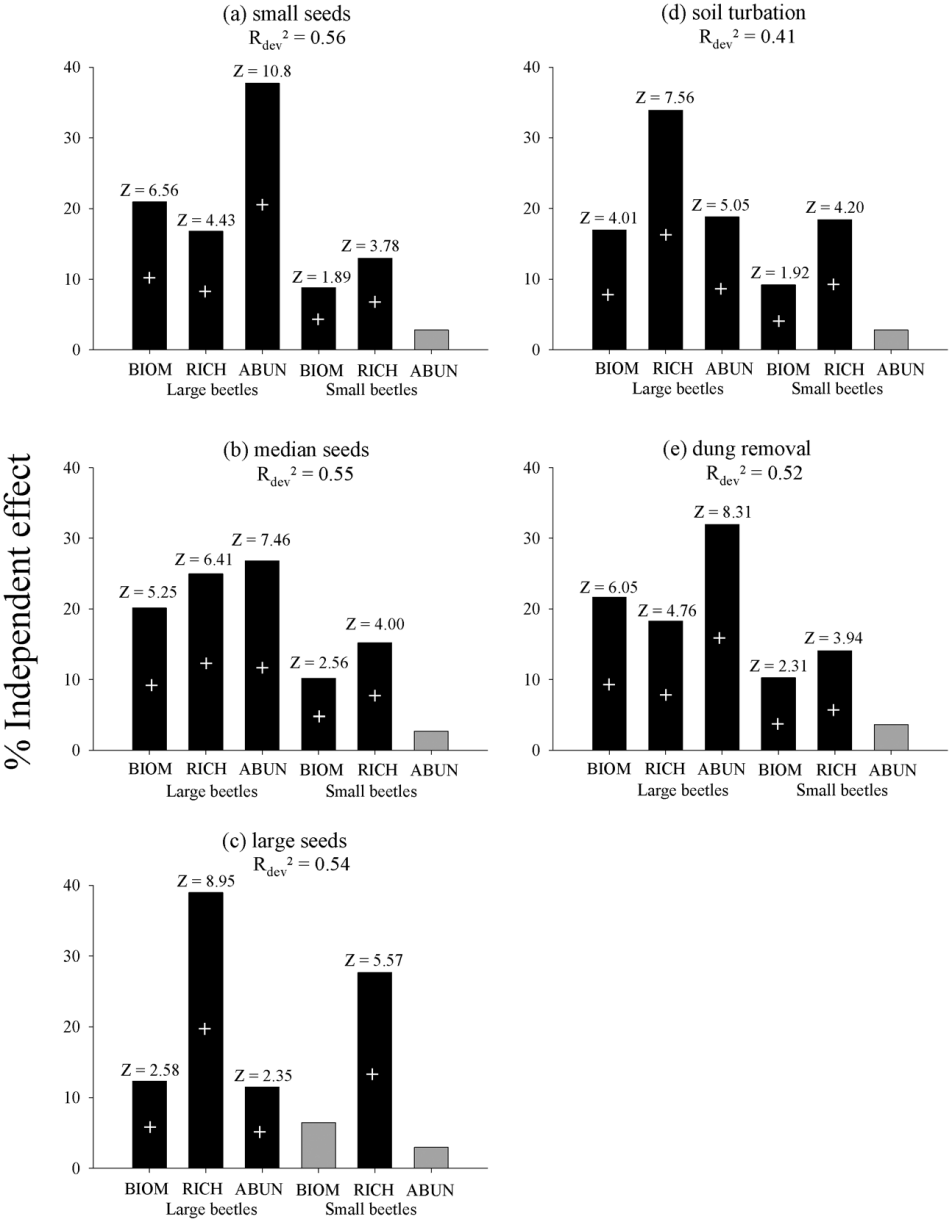
Analysis of hierarchical partitioning. Distribution of the percentage of independent effects of dung beetle community attributes (richness, abundance and biomass) on the amount of ecological function performed, as determined by hierarchical partitioning. Black bars represent significant effects (p<0.05) as determined by randomization tests. Positive relationships are shown by a+symbol. *R^2^_dev_* is the total deviance explained by a generalized linear model including the six predictive variables. Shown are: biomass of large beetles (BLB), richness of large beetles (RLB), abundance of large beetles (ALB), biomass of small beetles (BSB), richness of small beetles (RSB) and abundance of small beetles (ASB).

The results of the hierarchical partitioning performed to examine the independent effects of the six variables derived from combining the three community attributes for small- and large-beetle assemblages on the ecological functions measured, showed that, although all community attributes had a significant positive effect on all three ecological functions (except the abundance of small beetles on all ecological functions and the biomass of small beetles on the dispersal of large seed mimics), species richness and abundance of large beetles were the attributes with the highest explanatory values (Figure 5). Models including the six predictive variables explained between 41% and 56% of the response variable variance.

## Discussion

### Habitat Disturbance: Responses of Community Attributes Versus Community Functions

Three community attributes (species richness, abundance and biomass) and the three ecological functions (dung removal, soil excavation and secondary seed dispersal) were negatively affected by habitat disturbance. However, although some studies have found that some types of secondary forests and/or agroforests are able to maintain high values for some dung beetle community attributes [19], in the present study these land-use systems had significantly impoverished dung beetle communities and ecological functions (see also [43] and [44] for similar results). Larger-bodied dung beetles are more susceptible to abundance decline in disturbed systems [8], and these species are the most related to function loss [45], [25], [51].

In terms of dung beetle community attributes and dung removal, agriculture sites were more similar to agroforests and secondary forests than to pasture sites. This could be due, in part, to the agricultural areas having many tourist species (50% of all species in agriculture are singletons) which increase total species number. The high abundance and biomass of this system was due mainly to one small species (*Pseudocanthon*.aff. *xanthurum* (Blanchard, 1845), which was very abundant (85% of all individuals). Our agriculture sites were of small size (<1.5 ha) and were surrounded by forested habitats, which might be acting as sources of colonizing individuals, as has been shown to occur for forest fragments [46], [47], [48]. High abundance in these systems can also be explained by the fact that indigenous people remain longer in areas of agriculture and agroforestry, growing and harvesting food products for consumption, and defecating nearby, therefore providing a stable source of food supply for dung beetles [31]. These results are mirrored by ecological function parameters: in our study, many small beetles collectively were able to remove large amounts of dung from the agriculture system; however, small beetles build smaller tunnels than do large beetles, therefore excavating less soil. Larger dung beetles also bury more seeds than do smaller beetles [49]. The observed similar amounts of dung buried in agriculture and in forested systems (secondary forest and agroforest) could be explained by the compensatory density [50] of small beetles. Despite the large-beetle assemblage being the major group responsible for ecological functions, the small-beetle assemblage also proved important for the function of dung removal, though not in the case of the other ecological functions.

Unlike community attributes (species richness, abundance and biomass) and dung removal, the values for soil excavation and secondary dispersal of seed mimics in agriculture plots were more similar to those found in pasture. This highlights important changes in the dung beetle community in the agriculture plots, related to the loss of large species, which although often functionally more important than smaller species, are also more extinction prone [51].

These results clearly demonstrate the relevance of empirically estimating the amount of ecological functions rather than deducing it from community attributes, as has already been pointed out by previous studies [26], [52]. Furthermore, among the functional variables, dung removal was the least sensitive to habitat disturbance. However, dung removal is the functional variable most often measured because most ecological functions of dung beetles are a consequence of, and thus directly related, to dung removal, and because it is easily measured. Slade and collaborators [25] verified that the proportion of seeds removed covaried with the amount of dung removed, but that more small seeds were removed than large seeds for a given proportion of dung removed. Although a correlation was recorded in our study between dung removal and the other two ecological functions, our results show that dung removal might not accurately reflect the effect of habitat disturbance on other ecological functions, such as secondary seed dispersal.

### Prediction of Community Function through Community Attributes

Habitat disturbance indirectly affects the ecological functions of the dung beetle community by affecting one or more of their community attributes (richness, abundance and biomass) associated mainly with large species. Our results show that the variation in ecological functions was explained by changes in the three community attributes we measured. However, it should be mentioned that models including the six predictive variables (from large and small species) failed to explain 44–59% of the variation in the amount of ecological function recorded (Figure 4). Although our results demonstrated that the loss of large species can influence the loss of ecological functions performed by dung beetles, the loss of functions cannot be explained by species size alone. Thus, other factors are related to beetle size in terms of the loss of ecological functions, such as functional groups [23] and environmental variables.

Many studies have highlighted the important relationships between community attributes and ecological functions, but often emphasize the role of beetle abundance and biomass over species richness (e.g. [10], [14], [25], [53]). As already mentioned, large beetles remove and bury larger amounts of dung than do small beetles [54]. In our study system, species richness of large beetles was the variable that best explained the variation in the amount of dispersal for large seed mimics and the amoung of soil excavation. Similarly, the abundance of large beetles was the variable that best explained the variation in the amount of dispersal for small and medium seeds mimics, as well as in dung removal. In our study, the abundance of small beetles did not influence significantly any of the ecological functions measured, in accordance with [25], who showed that small beetles have little effect on dung and seed removal, although there is complementarity among different functional groups for better ecological function accomplishment. Thus, it is likely that higher species richness is related to a higher diversity of functional groups, an attribute that has been shown to be important in predicting the amount of ecological functions [23], [25], [45].

Species richness can be measured at least at two different levels: (i) the overall community species richness (which is the total number of species present in a study site), and (ii) the mean species richness at the level of sampling point (one or more traps located in close proximity), which is the mean number of species that is attracted to a single point in space and/or time (can also be referred to as species density, see [55]). The former metric of species richness is the one that is usually reported in community studies and, consequently, the one that is usually associated with ecological function. However, we believe that, for dung beetles, the second metric is more useful for the purpose of relating richness to function, because the functions occur at the level of individual defecations [26], [56]. For future studies designed to correlate number of species with the amount of any ecological function, we suggest using species density measured at the same spatial and temporal scales used to measure the function.

### Assessment Method for Estimating the Ecological Functions of Dung Beetles

As with any experimental manipulation, the method proposed might not be adequate when the purpose of a study is to quantify accurately the amount of an ecological function performed, because the arena fence and other manipulations might alter the normal dung-relocating behavior of some beetle species. In particular, for studies focusing on the secondary dispersal of seeds by dung beetles, other methodologies might yield more accurate and realistic results (e.g. [26,57). However, when the purpose of a study is to obtain an estimate of the amount of ecological function with the objective of comparing sampling points (distributed either in space and/or time), then we believe that our method is useful in adding a functional dimension to dung beetle community studies, particularly when the effects of habitat disturbance are being assessed. This is particularly useful because it can simultaneously estimate the amount of at least three different ecological functions. As discussed above, this is important because, although most functions show a high correlation with dung removal, such correlation is not perfect and the measurement of additional functions adds valuable information.

Although the amount of dispersal of small and medium seed mimics in disturbed forests was intermediate between that of primary forest and open areas (agriculture and pasture), the results for large seed mimics told a different story. For large seed mimics, dispersal was equally low in secondary forest, agriculture and pasture, but was higher in agroforest, suggesting that dispersal of large seeds might suffer even in habitats that maintain a relatively complex vegetation structure, such as secondary forests. In our study site we observed that loss of large species occurs in secondary forest; this might negatively impact secondary seed dispersal, particularly of large seeded-species. This can have important consequences for tropical forest regeneration and succession because, in general, large seeds are characteristic of primary forest tree species. This highlights the relevance of including seeds of different sizes when quantifying seed dispersal by dung beetles.

Based on our results, we have provided conclusions on the dung beetle community and their ecological functions, including secondary seed dispersal quantities [34], in different land-use systems. However, the process of seed dispersal until the establishment of the seedling depends on several factors. The effective dispersal of a seed depends on its size [32], the amount of dung in which it is embedded [35], the time of deposition, the season [26], and the size [14], [58] and composition of the dung beetle guilds [25]. The seed-to-seedling process can also vary on the depth at the seed is buried [32], [57] and the ability of the seed to avoid density-dependent factors [59], [60].

## Supporting Information

Table S1 Results of the analyses with generalized linear models (GLMs) showing the effects of land use systems on community attributes and ecological functions.
(DOCX)
