# Supplementary material for: Dung Beetle Community and Functions along a Habitat-Disturbance Gradient in the Amazon: A Rapid Assessment of Ecological Functions Associated to Biodiversity
Source: PLoS One. 2013 Feb 27;8(2):e57786. doi: 10.1371/journal.pone.0057786 (PMC3583983; doi:10.1371/journal.pone.0057786)
Supplement: Table S1 — Results of the analyses with generalized linear models (GLMs) showing the effects of land use systems on community attributes and ecological functions. (DOCX) [file pone.0057786.s001.docx]

| **COMMUNITY ATRIBUTES** | **Test value** | **P** |
| --- | --- | --- |
| **All dung beetles** |  |  |
| Abundance | F = 26.78 | < 0.001 |
| Richness | χ^2^ = 241.52 | < 0.001 |
| Biomass | F = 34.76 | < 0.001 |
| **Large** **dung** **beetles** |  |  |
| Abundance | F = 63.21 | < 0.001 |
| Richness | F = 62.76 | < 0.001 |
| **Small** **dung** **beetles** |  |  |
| Abundance | F = 56.77 | < 0.001 |
| Richness | F = 35.63 | < 0.001 |
| **ECOLOGICAL FUNCTIONS** | | |
| Dung removal | F = 17.84 | < 0.001 |
| Soil excavation | χ^2^ = 64.71 | < 0.001 |
| Dispersal of small seed mimics | F = 21.00 | < 0.001 |
| Dispersal of medium seed mimics | F = 31.9 | < 0.001 |
| Dispersal of large seed mimics | F = 22.5 | < 0.001 |

**Table S1.** Results of the analyses with generalized linear models (GLMs) showing the effects of land use systems on community attributes and ecological functions.
